# Eyes can tell: Assessment of implicit attitudes toward AI art

**DOI:** 10.1177/20416695231209846

**Published:** 2023-10-30

**Authors:** Yizhen Zhou, Hideaki Kawabata

**Affiliations:** Global Research Institute, 12869 Keio University, Tokyo, Japan; Department of Psychology, 12869 Keio University, Tokyo, Japan

**Keywords:** AI art, aesthetic evaluation, visual attention, creativity, eye-tracking

## Abstract

Advances in artificial intelligence (AI) have significantly improved the abilities of machines. Human-unique abilities, such as art creation, are now being challenged by AI. Recent studies have investigated and compared people's attitudes toward human-made and AI-generated artworks. These results suggest that a negative bias may exist toward the latter. However, none of these previous studies has examined the extent of this bias. In this study, we investigate whether a bias against AI art can be found at an implicit level. Viewers’ attitudes toward AI art were assessed using eye-tracking measures and subjective aesthetic evaluations. Visual attention and aesthetic judgments were compared between artworks categorized as human-made and AI-made. The results showed that although it was difficult for individuals to identify AI-generated artwork, they exhibited an implicit prejudice against AI art. Participants looked longer at paintings that they thought were made by humans. No significant effect of categorization of paintings was found in subjective evaluations. These findings suggest that although human and AI art may be perceived as having similar aesthetic values, an implicit negative bias toward AI art exists. Although AI can now perform creative tasks, artistic creativity is still considered a human prerogative.

Advances in artificial intelligence (AI) have led to the increasing use of machines in various tasks previously performed solely by humans or requiring human assistance. Even the creation of art, a capability previously considered unique to humans, is now being challenged by AI. Many text-to-image AI generators have been developed using generative adversarial networks (GANs) or diffusion models. Generative adversarial networks and diffusion models are generative AI that differ in training methodologies. Generative adversarial networks use an adversarial game between two neural networks (i.e., a generator and a discriminator) to generate image samples (Goodfellow et al., 2014), while diffusion models employ an iterative diffusion process to transform noise into data (Ho et al., 2020). Recently, it has been suggested that diffusion-based systems outperform GANs (Dhariwal & Nichol, 2021); they provide fine-grained control over the generation process, enabling users to manipulate the quality and diversity of the generated data. The training procedure of diffusion models is considerably more stable than that of GANs. Disco Diffusion is one of the most popular generators based on diffusion models. It is available as an open-source program and can be run on Google's Colaboratory online service. The generative system can synthesize paintings simply based on the text input given (known as the “prompt”). Traditionally, the creation of paintings requires mature skills and rich experience to accurately portray painters’ imaginations. However, even amateurs without technical expertise can now easily create visual art in collaboration with text-to-image AI generators. Given its powerful capabilities, AI visual art is already a mainstream phenomenon. In fact, without revealing its authorship, an AI-generated painting created by diffusion models has won art awards (Roose, 2022).

The popularity of AI-generated art has also fostered an interest in studying people's attitudes toward AI art. It is an important research direction for understanding beauty and creativity. Beauty as a subjective experience has long been debated, as with “beauty is in the eye of the beholder.” Recently, the idea that both objective and subjective factors modulate aesthetic experience has been strengthened (e.g., Leder & Nadal, 2014). The subjective experience of individuals plays a vital role in their aesthetic appreciation in addition to low-level visual features such as color or spatial properties. For instance, viewing artwork in a museum enhances the evaluation and memory of the artwork, as opposed to viewing it in a laboratory (Brieber et al., 2015; Pelowski et al., 2017). This suggests that nonsensory prior information may influence the aesthetic experience. Therefore, one may assume that knowing that an artwork is created by AI before viewing it influences viewers’ evaluations and preferences. Previous research has demonstrated that the attributional authorship of the artwork does significantly influence the preference and evaluation of viewers. For instance, participants appreciated artworks less when they believed the authors were not humans (Ragot et al., 2020). Participants also rated paintings as less appealing when they were labeled as computer-generated rather than declared as borrowings from an art gallery, even when the paintings were identical (Kirk et al., 2009). However, there is also evidence against the negative bias toward AI art when the authorship was manipulated by preassignment (Gangadharbatla, 2022; Hong & Curran, 2019). Gangadharbatla (2022) reported that they failed to find direct evidence that a negative bias existed for AI-generated art solely from authorship attribution knowledge. In Hong and Curran's (2019) study, attributing an artwork to AI did not influence the aesthetic evaluations of the artwork. Interestingly, they found that aesthetic value ratings were significantly influenced by the real authorship of the artwork (i.e., whether it was created by humans or AI). However, it should also be noted that only abstract paintings were evaluated in their study.

Previous studies have also examined the ability of participants to distinguish between human- and AI-made artworks. The results showed a consistent trend that people had difficulty discriminating whether humans or AI had created the artwork (Chamberlain et al., 2018; Gangadharbatla, 2022; Ragot et al., 2020). Interestingly, although the discrimination was difficult, some found an explicit prejudice against AI-associated artworks: the perceived aesthetic values of the artworks that participants categorized as AI-generated were lower than those of human-made artworks (Chamberlain et al., 2018). This work also reported that the order of categorization and rating tasks did not modulate the bias, as aesthetic values perceived by viewers may have been obtained prior to provenance categorization. The accuracy of judging whether the artwork is created by humans or AI also differs between abstract and representational artworks. Although overall, participants were biased to respond that artwork was created by humans rather than AI, they identified the representational images much more frequently as human-made than a chance level (i.e., 50%), while it was about the change level that the abstract ones were chosen to be created by humans. This indicates a bias that people tend to believe that representational images were created by humans.

Feelings regarding AI-associated art may be more complex than previously assumed. It has been reported that humans show mixed feelings toward AI technology: although people expressed positive attitudes in self-report questionnaires, they showed negative attitudes toward AI in an implicit association task (Fietta et al., 2022). Therefore, people's explicit and implicit attitudes toward AI art may be different or even opposite. Moreover, most previous studies investigating the difference in attitudes toward human- and AI-made art are survey studies using subjective questionnaires, and many were performed online. The extent to which this bias is observed remains unclear. Specifically, whether this bias can be reflected implicitly by behavioral measurements has not been investigated. Therefore, approaches other than self-reported judgments are needed to confirm an implicit bias toward AI art. Despite the importance of eye movements in visual perception research, including aesthetics, none of the previous studies have investigated attitudes toward AI art using the eye movements associated with participants’ self-reported aesthetic evaluations.

Because the aesthetic experience begins with a visual scan of the artwork, the measurement of eye movements is a very useful tool for investigating the aesthetic experience of visual artwork. It helps reveal the perceptual and cognitive processes underlying the perception and evaluation of visual artwork (Locher, 2006). Viewers tend to focus their gazes on specific regions of the visual images rather than viewing them randomly (Mackworth & Morandi, 1967). The measurement of eye movements, such as the density of fixations, can be interpreted as an index of overt selection that represents the viewer's interest in the observed image (Henderson & Hollingworth, 1999; Rizzolatti et al., 1987). For example, eye-tracking studies have repeatedly demonstrated that visual attention is associated with the attractiveness of faces (e.g., Leder et al., 2016; Maner et al., 2007; Mitrovic et al., 2016; Valuch et al., 2015). Particularly for aesthetic appreciation of art, a relationship between visual attention and aesthetic experience has been found: the more participants liked the artwork, the longer they looked at it (Brieber et al., 2014).

This study attempts to further explore the negative bias toward AI art. In particular, it aims to determine whether an implicit negative bias toward AI art exists. Viewers’ attitudes toward AI artwork were assessed implicitly and explicitly regardless of attributional authorship (i.e., the prior knowledge of authorship was not manipulated). First, we used a free-viewing paradigm to examine the bias in visual attention, which was measured by the total fixation duration (TFD), fixation count (FC), and mean fixation duration (MFD). Second, the subjective evaluations of aesthetic appreciation were assessed in six dimensions: beauty, liking, emotional valence, emotional arousal, familiarity, and concreteness. For emotional valence, because Menninghaus et al. (2019) suggested that measures for aesthetic emotions should include separate unipolar ratings for positive and negative response dimensions rather than bipolar scales, we used a two-dimensional evaluative scale (i.e., both positive and negative) instead of a simple bipolar scale ranging from unpleasant to pleasant. Each participant then completed a categorization task to identify whether humans or AI made the artwork. Before the experiment, participants were only instructed to view and rate “paintings” without being told some of them had been made by AI. Based on the findings of previous research, we expected a negative bias toward the paintings that were categorized as AI-made. We hypothesized that the gaze behaviors and subjective evaluations would significantly differ between paintings that participants had selected as human- and AI-made. We also compared the accuracy of provenance categorization between the AI-generated and human-made paintings and predicted that participants would perform poorly in identifying AI-made representational paintings.

## Method

### Participants

Thirty-four Japanese participants (mean age = 21.3 years; standard deviation [SD] = 1.16 years; 22 women) naive to art criticism participated in the experiment. Participants were undergraduate students recruited from universities located in the Greater Tokyo Area. They were compensated with 1,100 Japanese yen for their efforts. All the participants had normal or corrected-to-normal vision. This experiment was approved by the local Ethics Committee of Keio University (approval number: 220020000). Written informed consent was obtained from all the participants in advance.

### Materials

#### Stimuli

We selected 20 landscape paintings from The Vienna Art Picture System dataset (Fekete et al., 2022, a full list of paintings can be found in Table A1 in Appendix A). The AI paintings were created using an open-source program, Disco Diffusion (https://github.com/alembics/disco-diffusion, accessed on November 17, 2022). The prompts were based on the titles, descriptions, and authors’ styles (see Table 1 for all the prompts). All stimuli were shown on a light-gray background. To normalize the objective properties of each individual painting on aesthetic appreciation, we used the MATLAB Image Processing Toolbox (MATLAB, 2021a, The MathWorks, Inc.) to quantify the paintings’ color according to the hue, saturation, and brightness representations of the RGB color model. Hue describes the dominant wavelength of a color. Saturation refers to the intensity or “colorfulness” of a given color. Brightness refers to the brightness of a given color. The average values of all these three dimensions were calculated for all pixels in each painting. In addition, the entropy values of each painting were also computed using the same MATLAB toolbox. Entropy refers to the level of “randomness” (i.e., the level of disorder) of the pixels in a painting. Table 2 lists the mean values of each objective property (i.e., hue, saturation, brightness, and entropy) for all paintings. A paired-sample *t*-test was conducted to confirm that human- and AI-made paintings do not differ in objective properties. No significant difference was found between human- and AI-made paintings for hue (Mean: 0.30 vs. 0.31), *t*(19) = −0.482, *p* = .64; saturation (Mean: 0.39 vs. 0.38), *t*(19) = 0.380, *p* = .71; brightness (Mean: 0.53 vs. 0.55), *t*(19) = −0.436, *p* = .67; and entropy (Mean: 7.46 vs. 7.29), *t*(19) = 1.006, *p* = .33.

**Table 1.** Prompts for Generating AI-made Paintings.

Prompts
Prompt 1: “A Street”, “Giorgia ÒKeeffe”
Prompt 2: “After Sir Christopher Wren”, “Charles Demuth”
Prompt 3: “A high nave with the Gothic windows can be seen between the surrounding houses”, “Lyonel Feininger”
Prompt 4: “Boats are returning to the shore at sunset”, “Max Pechstein”
Prompt 5: “Café Terrace at Night”, “Vincent van Gogh”
Prompt 6: “The rooftops of Collioure”, “Henri Matisse”
Prompt 7: “Fishing boats at Collioure”, “André Derain”
Prompt 8: “Harbor of Bordeaux”, “Édouard Manet”
Prompt 9: “Houses at Night”, “Karl Schmidt-Rottluff”
Prompt 10: “Landscape of mountains and trees”, “Paul Gauguin”
Prompt 11: “The picture is a landscape, illuminated by a grayish-violet unearthly light. Against the background of the desert space, there are a cactus, beans, a yellow figure and a peculiar shaggy stick. A dark cloud occupying the right side of the canvas hangs menacingly over the field”, “Yves Tanguy”
Prompt 12: “Palace of the Popes at Avignon”, “Paul Signac”
Prompt 13: “Pines along the shore”, “Henri Edmond Cross”
Prompt 14: “Splashes of sunlight on the terrace”, “Maurice Denis”
Prompt 15: “Steel mill near Charleroi”, “Maximilien Jules Luce"
Prompt 16: “Quiet day by the sea”, “Lyonel Feininger”
Prompt 17: “Terrace in Meudon”, “Paul Signac”
Prompt 18: “The Sacred Grove”, “Arnold Böcklin”
Prompt 19: “The Seine at Herblay”, “Maximilien Jules Luce”
Prompt 20: “Village on the Sea”, “Karl Schmidt-Rottluff”

**Table 2.** Mean Values for Objective Properties (Nos. 1−20: Human-made Paintings; Nos. 21−40: AI-made Paintings).

No. of painting	Hue	Saturation	Brightness	Entropy
1	0.330	0.206	0.451	7.691
2	0.139	0.126	0.687	7.488
3	0.186	0.560	0.394	7.528
4	0.292	0.410	0.368	7.502
5	0.347	0.504	0.550	7.779
6	0.407	0.331	0.742	7.565
7	0.259	0.398	0.609	7.428
8	0.220	0.324	0.557	7.717
9	0.305	0.678	0.429	7.107
10	0.283	0.536	0.536	7.545
11	0.202	0.119	0.464	6.931
12	0.426	0.295	0.696	7.387
13	0.434	0.330	0.744	7.706
14	0.134	0.818	0.555	7.044
15	0.400	0.133	0.373	7.287
16	0.406	0.389	0.585	7.685
17	0.464	0.346	0.703	7.786
18	0.196	0.419	0.294	7.025
19	0.346	0.393	0.605	7.811
20	0.183	0.520	0.338	7.208
21	0.417	0.400	0.491	7.494
22	0.090	0.286	0.719	7.664
23	0.291	0.218	0.558	7.163
24	0.393	0.383	0.666	7.370
25	0.189	0.666	0.171	5.019
26	0.450	0.336	0.717	6.747
27	0.217	0.495	0.636	7.440
28	0.357	0.192	0.553	7.777
29	0.267	0.546	0.361	5.768
30	0.189	0.599	0.544	7.808
31	0.382	0.272	0.588	7.791
32	0.705	0.269	0.603	7.457
33	0.273	0.365	0.617	7.745
34	0.156	0.313	0.561	7.300
35	0.341	0.332	0.394	7.594
36	0.299	0.368	0.642	7.462
37	0.367	0.228	0.679	7.547
38	0.339	0.483	0.335	7.446
39	0.281	0.291	0.530	7.916
40	0.212	0.492	0.592	7.287

Paintings were rescaled to a maximum width of 1,200 pixels while maintaining their original aspect ratio, resulting in heights ranging from 784 to 900 pixels. Photoshop was used to remove the artists’ signatures from the paintings.

#### Apparatus

The experiment was conducted in a dimly lit room. A screen-based eye tracker (300 Hz, 9-point calibration and validation, Tobii Pro Spectrum, Tobii Pro AB) was used to track the movements of both eyes of each participant. The images were presented on a 23.8″ IPS, gamma-corrected monitor (FlexScan EV245, EIZO Corporation) at a distance of 63 cm. Participants’ heads were rested on a chin rest to minimize head movement. The presentation of the stimuli was created and run by an open-source toolbox Titta (Niehorster et al., 2020). This toolbox allowed us to control the eye tracker by Python, while the eye movements were recorded using the Tobii Pro Lab software (Tobii Pro AB).

### Procedure

**Figure 1.**
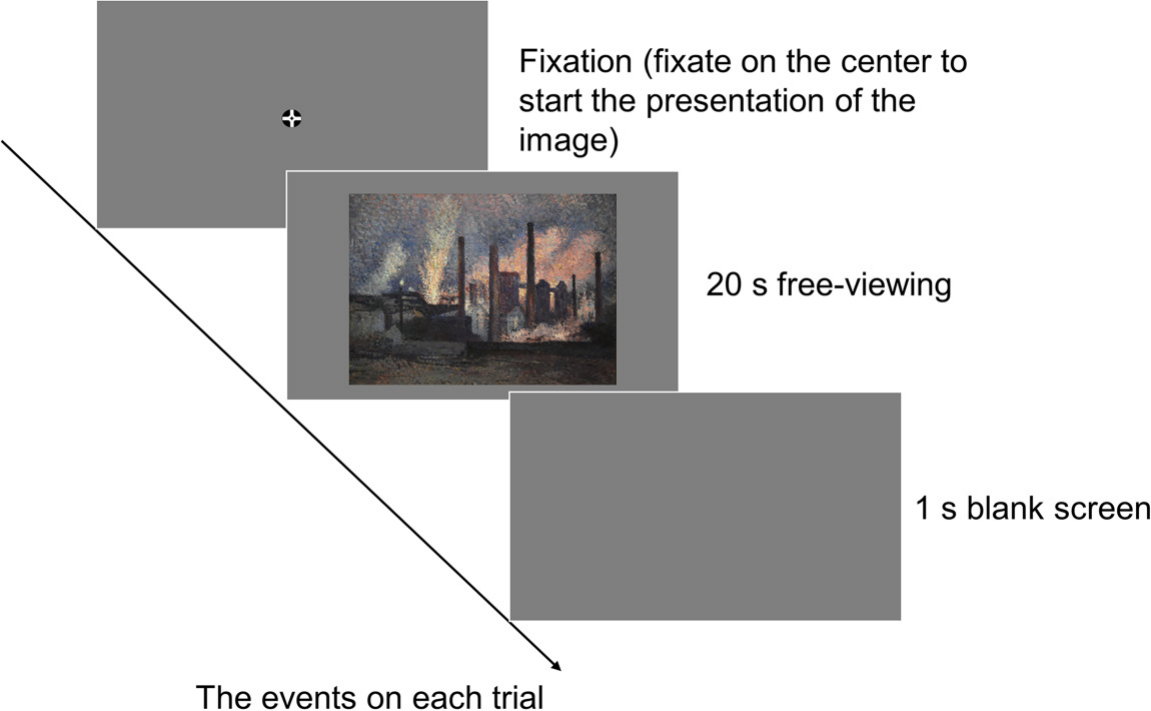
A schematic representation of the events on each trial. The painting shown in this figure is a copyright-free image sourced from WikiArt (http://www.wikiart.org/).

This study involved three tasks. In the first task (the free-viewing task, see Figure 1), 20 human-made and 20 AI-generated paintings were presented on the screen. The task began with the calibration of the eye tracker, and participants completed two practice trials using two representational paintings (these paintings were not shown again during the following main task). All stimuli were presented in a random sequence, and each painting was displayed on the screen for 20 s, followed by a 1 s blank screen. Between each trial, participants had to fixate on the fixation cross that appeared in the middle of the screen to proceed to the subsequent trial. The participants were instructed to recalibrate if this fixation did not work. During the presentation of the painting, participants viewed it freely. A break was given for every 20 stimuli, and recalibration was required after the break. The entire task took approximately 20 min.

After the free-viewing task, the participants completed a subjective rating task for all stimuli. In this task, all paintings were shown in random order on the same monitor used in the free-viewing task. Participants evaluated each painting for its beauty, liking, valence (both positive and negative), arousal, familiarity, and concreteness using a visual analog scale (maximum scale value: 0–100). For example, for the rating of beauty, the left end was labeled as “very unbeautiful” and the right as “very beautiful.” The participants responded by dragging the mouse and pressing the space key once they had decided to proceed to the next trial. No time limit was set for the responses. It took approximately 20 min to complete this task.

Finally, the participants performed a categorization task to identify whether the paintings they had viewed and rated were made by humans or AI. The participants performed this task on the same monitor by responding to a two-alternative forced choice. All stimuli were presented in random order.

### Data Analysis

The results of 1,360 trials with 34 participants and 40 paintings in the free-viewing task were analyzed. The fixation metrics of each participant provided by the Tobii Pro Lab software were used in the analysis. All fixations on the paintings were detected by Tobii velocity-threshold identification fixation filter (as described by Salvucci & Goldberg, 2000 and Komogortsev et al., 2010). We conducted a linear mixed model (LMM) analysis on fixations. The lmer function in the R package lmerTest (Kuznetsova et al., 2017) was used to generate the LMM.

The TFD, which is the dwell time during the total viewing period for each painting in milliseconds), FC for each painting, and MFD in milliseconds were used as dependent variables. An LMM analysis was run for each dependent variable. The independent variables include the authors of paintings (human- or AI-made) and the participants’ categorization of the authorship. To visualize the fixation results, heatmaps that coded the average sums of fixation durations for each painting were created by Tobii Pro Lab using different colors (red indicates the longest time of fixation within certain areas of the stimulus, and green the least, with varying levels in between). All paintings, together with their heatmaps, can be found in Figure S1−40 in the Supplementary Material.

The results of the trials for the subjective rating task completed by the participants were analyzed. Similarly, we ran an LMM analysis for each dependent variable (i.e., scores of beauty, liking, valence [positive and negative], arousal, familiarity, and concreteness). The independent variables include the authorship and painting categories. Table 3 shows the descriptive statistics of all subjective ratings.

**Table 3.** Mean Values for Subjective Ratings (Nos. 1−20: Human-made Paintings; Nos. 21−40: AI-made Paintings).

No. of painting	Beauty	Liking	Valence (positive)	Valence (negative)	Arousal	Familiarity	Concreteness
1	46.38	51.71	32.29	60.38	36.24	17.03	44.32
2	70.56	71.68	61.97	40.65	51.03	15.26	55.50
3	54.94	54.88	45.35	46.76	47.59	12.50	25.26
4	41.00	43.24	36.03	59.62	46.97	12.71	46.79
5	86.09	86.65	79.44	21.06	65.00	67.50	72.94
6	59.38	54.21	71.18	23.35	41.74	16.65	20.59
7	47.00	42.15	59.91	37.94	39.97	15.56	16.76
8	68.59	68.88	49.15	48.82	50.47	23.56	85.76
9	35.38	37.21	33.03	60.03	40.32	10.65	13.74
10	80.56	77.47	80.24	19.91	51.97	30.03	43.94
11	45.71	51.53	34.12	62.12	48.09	11.97	29.24
12	83.97	76.24	79.38	19.38	56.18	27.12	39.12
13	77.03	67.35	75.03	25.91	49.26	27.26	42.18
14	35.82	35.79	26.15	62.65	59.12	5.32	11.26
15	56.26	54.91	24.06	67.00	42.97	19.15	50.38
16	73.68	69.44	58.50	41.50	56.79	16.71	15.88
17	84.41	78.21	83.91	14.74	58.47	28.15	48.03
18	81.06	72.94	37.53	63.41	62.88	11.59	77.91
19	83.56	76.35	82.97	21.32	59.65	28.24	40.65
20	23.74	28.15	32.00	55.03	30.85	13.09	23.29
21	54.41	58.21	31.53	62.24	44.21	10.94	44.38
22	55.97	59.00	37.26	46.00	39.12	9.74	50.03
23	49.06	49.68	25.18	65.74	38.94	14.74	53.44
24	54.97	53.44	53.47	35.35	37.85	13.74	32.68
25	78.85	79.94	51.26	50.68	70.21	24.15	62.38
26	39.00	37.82	46.82	37.82	41.65	9.59	13.97
27	34.41	33.29	35.88	53.24	38.09	15.53	31.26
28	74.32	75.00	45.47	53.76	51.44	19.15	73.35
29	33.41	33.71	22.38	72.44	53.50	7.76	14.12
30	63.65	60.06	54.76	41.21	37.85	16.59	51.71
31	47.62	49.00	26.12	69.12	47.00	11.44	24.29
32	54.15	51.88	49.50	40.79	35.44	11.32	52.09
33	55.68	53.38	54.94	46.06	41.62	15.06	57.00
34	66.62	61.24	65.32	32.35	45.85	14.35	37.18
35	65.41	63.65	42.68	51.47	50.06	17.50	79.32
36	58.88	59.03	39.76	52.00	44.53	15.03	32.38
37	63.82	57.41	56.97	43.15	38.41	17.38	50.00
38	70.09	66.00	48.21	49.76	52.35	14.09	60.09
39	75.79	74.74	54.06	38.18	52.06	14.76	76.41
40	27.71	27.56	31.15	62.79	44.74	8.76	10.76

We also averaged each participant's correct responses on the categorization task to measure their performance. Categorization performance was then divided and compared using a one-factor analysis of variance for human- and AI-made paintings. All data generated or analyzed during the current study are included in the Research Data file.

## Results

### Free-Viewing Task

The LMM analysis was used to assess the fixed effects of the participants’ categorization of paintings and the actual authorship of paintings. For the free-viewing task, we constructed a full model (with fixed effects of the categorization and authorship of paintings) and compared the reduced models against them to specifically examine the significance of the categorization and authorship effects. We also included an interaction between the painting authorship and identification category to assess the relationship between actual authorship and participants’ judgments of authorship. Because the paintings varied in size, a random intercept of image size in the number of pixels was included in the LMM. However, including this random intercept did not improve the model's explanatory power. As a result, we included only random by-painting and by-participant intercepts to account for observational dependence.

For TFD (intercept: Estimate [Est.] = 13752.38, standard error [SE] = 478.37, all units are in milliseconds), there was no significant interaction between the paintings’ actual authorship and the participants’ belief in the authorship (likelihood ratio test comparing the full model and the reduced model lacking the interaction: χ^2^ = 1.74, *p* = .19). There was a significant difference between paintings categorized as human- and AI-made. The paintings that were chosen as human-created by participants had a longer TFD than the AI-generated paintings (χ^2^ = 5.84, *p* = .016): the viewing time increased by 330.68 ± 136.14 (SE). No significant effect was found for actual authorship (χ^2^ = 0.0026, *p* = .96).

For FC, human- and AI-created paintings did not differ significantly in FC (χ^2^ = 0.49, *p* = .48). The participants’ categorization also did not influence the FC (χ^2^ = 1.04, *p* = .31). No significant interaction was found between the actual authorship of paintings and participants’ subjective judgments (likelihood ratio test comparing the full model and the reduced model lacking the interaction: χ^2^ = 0.76, *p* = .38).

Similarly, no significant interaction was found for MFD (χ^2^ = 0.48, *p* = .12). The effects of paintings’ actual authorship and categorization of the paintings were also not significant (χ^2^ = 0.70, *p* = .41; χ^2^ = 2.82, *p* = .93).

### Subjective Rating Task

The LMM analysis was used to assess the fixed effects of the participants’ categorization of paintings and the author of the paintings. We constructed a full model (with fixed effects of categorizations and authorships of the paintings) and compared the reduced models against it to specifically examine the significance of the fixed effects on the subjective ratings of beauty, liking, valence (positive and negative), arousal, familiarity, and concreteness. In the full model, we also included random by-painting and by-participant intercepts to account for the observations’ dependency. The results showed that the main effects of categorization and authorship were not significant for all ratings: categorization (beauty; χ^2^ = 0.69, *p* = .40, liking; χ^2^ = 1.66, *p* = .20, valence [positive]; χ^2^ = .068, *p* = .021, valence [negative]; χ^2^ = 0.014, *p* = .91, arousal; χ^2^ = 0.0028, *p* = .096, familiarity; χ^2^ = 0.20, *p* = .65, concreteness; χ^2^ = 1.80, *p* = .18), and authorship (beauty; χ^2^ = 0.87, *p* = .035, liking; χ^2^ = 0.79, *p* = .37, valence [positive]; χ^2^ = 3.44, *p* = .064, valence [negative]; χ^2^ = 2.32, *p* = .13, arousal; χ^2^ = 2.16, *p* = .14, familiarity; χ^2^ = 3.71, *p* = .054, concreteness: χ^2^ = 0.69, *p* = .41).

**Figure 2.**
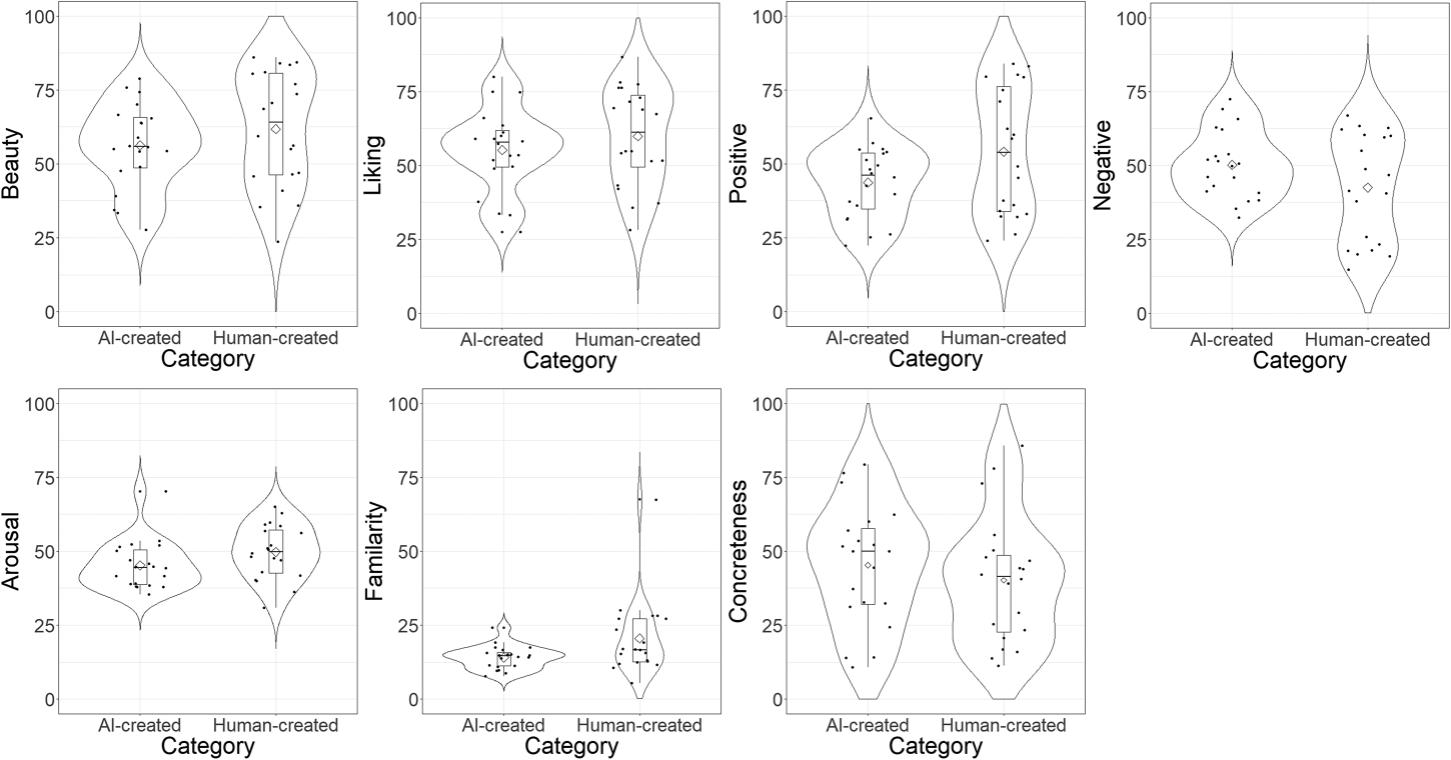
Violin plots showing the distribution of the participants’ average evaluations of beauty, liking, valence (both positive and negative), arousal, familiarity, and concreteness of the human- and AI-made paintings.

We also compared the distribution of the average ratings of beauty, liking, valence (both positive and negative), arousal, familiarity, and concreteness of the human- and AI-made paintings (see Figure 2). Although the paintings did not differ in subjective evaluations, one painting, Café Terrace at Night (Place du Forum in Arles) by Vincent van Gogh, was particularly familiar to participants.

### Categorization Task

The results of the categorization task indicate that human-made paintings were successfully detected with 68% accuracy, and AI-made paintings yielded a much lower accuracy rate of 43%. Accuracy for the two types of images was significantly different from one another: *F*_(38, 1)_ = 28.83, *p* < .001, η^2^ = 0.431.

## Discussion

This study investigated people's attitudes toward AI-generated artwork when no prior knowledge of the artwork's authorship was available. Specifically, we investigated the existence of an implicit negative bias toward AI art. In the present study, we examined and directly compared the behavioral and subjective measurements of paintings that participants categorized as human-created and AI-generated. To assess the behavioral responses during aesthetic appreciation, we asked the participants to explore the painting for 20 s in a free-viewing paradigm and measured their gaze behaviors. We found a negative bias toward AI art on TFD: the paintings categorized as made by humans increased the fixation duration by 331 ms. In other words, the paintings that participants categorized as made by humans were looked at longer than those that were categorized as AI-made. The results of subjective evaluations of the artwork indicate that such bias cannot be found explicitly at a subjective level: no significant difference was found between paintings categorized as human-made and AI-generated. The paintings that participants selected as human-made were not rated higher in subjective measures of aesthetic appreciation in terms of beauty, liking, valence, arousal, familiarity, and concreteness.

In line with previous research comparing attitudes toward human- and AI-made artworks (Chamberlain et al., 2018; Gangadharbatla, 2022; Ragot et al., 2020; Ueda et al., 2021), the participants were unable to accurately identify the authors of the paintings. We also found that human-made paintings were more likely to be categorized as human-made paintings, whereas participants performed poorly in identifying AI-generated paintings, which agrees with the findings of Chamberlain et al. (2018) and Gangadharbatla (2022). Moreover, both studies revealed that the type of artwork influenced the perception of the artwork. Participants were biased in responding that representational artworks were created by humans, whereas abstract artworks were less frequently assumed to be made by humans. Therefore, only representational paintings depicting landscapes were used in our study to exclude the effect of the types of painting.

The findings of this study suggest that people's explicit and implicit attitudes toward AI art may be divergent. It was hypothesized that the categorization of artworks influenced both gaze behaviors and subjective evaluations. However, no negative bias was found in subjective evaluations of paintings that participants categorized as AI-generated. This suggests that human and AI art were perceived as having similar aesthetic values, at least for people who were naive to art criticism. They did not consider AI-made paintings less beautiful, likable, or pleasing. This result is inconsistent with the previous report that the categorization of artworks influenced aesthetic perception that artworks categorized as human-made were always rated more aesthetically pleasing than computer-generated artworks, regardless of the order of the evaluation and categorization tasks (Chamberlain et al., 2018). In their study, both abstract and representational artworks were evaluated. In contrast, we have only tested figurative landscape paintings. Because abstract and representational paintings differ in visual characteristics, such as the distribution of oriented lines and shapes, this difference may modulate the negative bias in subjective evaluations toward AI art according to the types of paintings. The negative bias may be more robust for certain types of artworks, and hence it can be observed explicitly.

The results of the eye-tracking measures provide the first evidence that negative bias toward AI can be found at the implicit level. Visual attention to paintings differed between paintings that participants selected as human- and AI-made. Authorship categorization had a significant effect on the TFD; it increased the fixation duration by more than 330 ms during the free-viewing period of the painting. Participants tended to look longer at the paintings they selected as human-made in the categorization task. In conjunction with previous findings, our results indicate that a negative bias toward AI art can emerge implicitly during aesthetic appreciation.

Alternative to the implicit bias toward AI art, another possible factor could account for such an effect. Namely, the output properties of generative modeling may modulate this effect. Diffusion models rely on prompts to generate images, and it remains questionable whether such creation is “real” art because it lacks originality. According to Hertzmann (2018), art requires a desire to express something, but AI is unable to create “art” as it lacks this intentionality (or “soul”) and has no content to express. Although powerful AI generators such as diffusion models can make paintings very similar to human-made ones in a way that viewers are unable to detect the provenance alone from the content or surface characteristics of the paintings, it is possible that diffusion models produced distinctive but mildly irritating properties of the output. In other words, although the bias effect was abolished at the level of subjective evaluations, it was found at the level of eye-tracking measures because of the inconspicuously distinct features of generative modeling.

AI now challenges humans in many human-exclusive domains, including art and creativity, although AI-generated art cannot fully mimic human-made art. It is difficult for people to tell whether AI-generated artwork is produced by AI or humans. However, despite their superficial similarity, people seemed to prefer paintings that they assumed were created by humans. People's feelings about AI may be complex and mixed. It has been reported that the advancement of AI in recent decades has fueled widespread concern about its impact on certain aspects of society (Li & Huang, 2020). This fear may stem from the idea that AI will steal jobs and raise unemployment rates, or it may be connected to the fear of diversity. However, because artists only represent a small portion of the human population and are usually not considered “workers,” the fear of losing jobs may be less critical in the context of art creation. Moreover, our participants were naive to art criticism and not creators of artworks themselves; thus, they were not afraid that AI would take their jobs. Because we did not manipulate the authorship assignment, we did not directly assess whether the participants’ responses were influenced by fear of AI. Future research could investigate whether feelings of fear may induce the bias.

This study has several limitations. First, we used a specific image generator based on diffusion models to create paintings. Therefore, these results cannot speak to the broader comparison of paintings created by humans and computers, and the observed bias effect should be interpreted with respect to the current painting set but not as a “general” bias toward AI art. Second, the paintings were presented on a computer screen in a laboratory. The experience of viewing a painting in a museum cannot be wholly duplicated with a screen in a laboratory (Augustin & Wagemans, 2012; Brieber et al., 2015; Locher et al., 1999). Because visual perception is significantly more sophisticated and complex in the real world than in an experimental paradigm, our findings still need to be validated in the ecological setting of an art museum. Third, only Japanese students participated in this experiment. A recent study that used an online survey has reported cultural differences between Western and Eastern participants’ perceptions of human- and AI-made poems and paintings (Wu et al., 2020). The results of this study suggest that participants from the United States were more critical of AI-generated than human-created content. Chinese participants were generally more optimistic about AI-made content, although they also appreciated human-authored content more than AI-generated content. Future studies should investigate this bias across different cultures.

In summary, the present study assessed whether negative bias toward AI art was present among art viewers, as well as its extent. In the experiment, participants viewed and rated paintings made by humans or AI. Our results indicate an implicit bias toward AI art. Although participants were unable to identify whether the paintings were made by AI and evaluated human- and AI-made paintings equivalently in terms of perceived aesthetic values, they spent more time viewing the paintings they categorized as human-made than AI-generated. This finding suggests that a negative bias toward AI art can be reflected at an implicit level. Although AI is now capable of performing creative tasks typically undertaken by humans, artistic creativity is still considered a human-exclusive ability.

## Supplemental Material

sj-csv-1-ipe-10.1177_20416695231209846 - Supplemental material for Eyes can tell: Assessment of implicit attitudes toward AI art Click here for additional data file. Supplemental material, sj-csv-1-ipe-10.1177_20416695231209846 for Eyes can tell: Assessment of implicit attitudes toward AI art by Yizhen Zhou and Hideaki Kawabata in i-Perception

sj-csv-2-ipe-10.1177_20416695231209846 - Supplemental material for Eyes can tell: Assessment of implicit attitudes toward AI art Click here for additional data file. Supplemental material, sj-csv-2-ipe-10.1177_20416695231209846 for Eyes can tell: Assessment of implicit attitudes toward AI art by Yizhen Zhou and Hideaki Kawabata in i-Perception

sj-csv-3-ipe-10.1177_20416695231209846 - Supplemental material for Eyes can tell: Assessment of implicit attitudes toward AI art Click here for additional data file. Supplemental material, sj-csv-3-ipe-10.1177_20416695231209846 for Eyes can tell: Assessment of implicit attitudes toward AI art by Yizhen Zhou and Hideaki Kawabata in i-Perception

sj-7z-4-ipe-10.1177_20416695231209846 - Supplemental material for Eyes can tell: Assessment of implicit attitudes toward AI art Click here for additional data file. Supplemental material, sj-7z-4-ipe-10.1177_20416695231209846 for Eyes can tell: Assessment of implicit attitudes toward AI art by Yizhen Zhou and Hideaki Kawabata in i-Perception

## Appendix A

**Table A1.** List of Human-made Paintings.

Title	Artist	No.
A Street	Giorgia ÒKeeffe	1
After Sir Christopher Wren	Charles Demuth	2
Barfüßerkirche in Erfurt I	Lyonel Feininger	3
Boats Returning	Max Pechstein	4
Café Terrace at Night (Place du Forum in Arles)	Vincent van Gogh	5
Die Dächer von Collioure	Henri Matisse	6
Fishing Boats, Collioure	André Derain	7
Harbor of Bordeaux	Édouard Manet	8
House at Night	Karl Schmidt-Rottluff	9
Landscape	Paul Gauguin	10
Mama, Papa is Wounded!	Yves Tanguy	11
Palace of the Popes at Avignon	Paul Signac	12
Pines Along the Shore	Henri Edmond Cross	13
Splashes of Sunlight on the Terrace	Maurice Denis	14
Steel mill near Charleroi	Maximilien Jules Luce	15
Stiller Tag am Meer III	Lyonel Feininger	16
Terrasse de Meudon	Paul Signac	17
The Sacred Grove	Arnold Böcklin	18
The Seine at Herblay	Maximilien Jules Luce	19
Village on the Sea	Karl Schmidt-Rottluff	20
